# Validation of *ITPR2*, *DPF3*, *EPAS1*, and *PVT1*-associated SNPs as biomarkers for RCC in an independent case-control cohort

**DOI:** 10.3389/fmed.2026.1734511

**Published:** 2026-03-11

**Authors:** C. M. Morales-Álvarez, A. C. Jiménez-Domínguez, R. M. Rios-Pelegrina, E. Arance, F. Marín-Benesiu, F. Vázquez-Alonso, L. J. Martínez-González, M. J. Álvarez-Cubero

**Affiliations:** 1 Department of Biochemistry and Molecular Biology III, Faculty of Medicine, PTS Granada, University of Granada, Granada, Spain; 2 GENYO, Centre for Genomics and Oncological Research: Pfizer, University of Granada, Andalusian Regional Government, PTS Granada, Granada, Spain; 3 Department of Urology, University Hospital Virgen de las Nieves, Granada, Spain; 4 Department of Pathology, Hospital Clínico San Cecilio, Granada, Spain; 5 Biosanitary Research Institute, ibs.GRANADA, Granada, Spain

**Keywords:** biomarkers, *DPF3*, *EPAS1*, *ITPR2*, renal cell carcinoma, SNPs

## Abstract

**Introduction:**

Renal cell carcinoma (RCC) is a heterogeneous malignancy influenced by genetic and environmental factors. Previous genome-wide association studies (GWAS) have identified risk single nucleotide polymorphisms (SNPs) associated with RCC susceptibility, particularly within genes such as *ITPR2*, *DPF3*, *EPAS1*, *PVT1*, and *MYC*. These SNPs are in regions implicated in key cellular processes like calcium signaling, chromatin remodeling, hypoxia response and oncogenesis. These pathways are highly relevant to RCC pathogenesis, although the functional significance of these genetic variations in sporadic RCC remains insufficiently characterized.

**Methods:**

This study analyzed five GWAS-identified SNPs—rs1049380 and rs10771279 (*ITPR2*), rs4903064 (*DPF3*), rs7579899 (*EPAS1*), and rs35252396 (*PVT1/MYC*)—in a Spanish case-control cohort comprising 168 RCC patients and 259 healthy controls. Genotyping was performed from buccal swabs, and gene expression levels were assessed in 33 paired formalin-fixed paraffin-embedded (FFPE) tumor and adjacent normal kidney tissue samples. Associations between SNPs, overall survival, and expression of quantitative trait loci (eQTLs) were evaluated in relation to RCC risk and RCC progression in the case of survival curves.

**Results:**

The C/C genotype of *ITPR2* rs10771279 was nominally associated with a protective effect (OR: 0.41), with higher *ITPR2* expression observed in healthy tissues than in RCC. The C/C genotype of *DPF3* rs4903064 was nominally correlated with increased RCC risk (OR: 2.21) and higher *DPF3* expression, potentially linked to hypoxia-inducible pathways. Similarly, *EPAS1* rs7579899 A/A genotype was nominally associated with RCC risk (OR: 1.78) While *PVT1/MYC* rs35252396 did not show susceptibility relation, both genes showed upregulated expression in RCC tissue. In survival analyses, the G allele of rs1049380 (*ITPR2*) was significantly associated with reduced 5-year survival in metastic and non-metastatic patients. Additionally, the AC genotype of rs35252396 showed nominal associations with highest risk in 5-year survival models.

**Conclusion:**

This study provides independent evidence supporting the biological relevance of GWAS-identified loci in RCC. While several variants showed nominal associations with disease risk, *ITPR2* rs1049380 emerged as a variant of potential prognostic relevance for five-year overall survival. Overall, these findings highlight the differential contribution of genetic variants to RCC susceptibility and progression and should be considered hypothesis-generating, warranting validation in larger, independent cohorts.

## Graphical abstract

**Figure:**
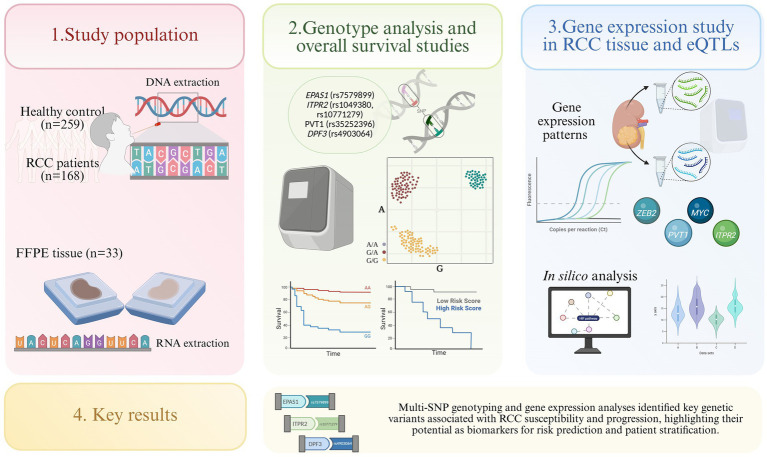
Created with BioRender.com.

## 1 Introduction

Kidney cancer was the 14th most diagnosed cancer worldwide in 2024. The annual incidence is 434,840 new diagnoses, accounting for 2.2% of all cancers, and an estimated number of deaths of 155,953 per year (1). In over 85% of kidney cancers, the pathological subtype is renal cell carcinoma (RCC). The median age at diagnosis is 64 years and is more common in males than females (a ratio of approximately 2:1) (2). The mainly histopathological subtype of RCC is clear cell RCC (ccRCC), reported in approximately 75% of patients, followed by papillary RCC type in 15% of patients, chromophobe RCC in 5%, and several rare RCC subtypes and unclassified RCC (3). Epidemiological studies have conclusively identified three modifiable risk factors: smoking, obesity body mass index and hypertension (4). Other risk factors may include physical inactivity, occupational exposure such as heavy metals and industrial solvents and a history of diabetes mellitus (5).

Having a first-degree relative with kidney cancer is also associated with an increased risk of RCC. Among RCC patients, 3.1% have one or more FDR (first-degree relative—parents and siblings) (6). Renal cancer can be related to an inherited or *de novo* monogenic germline alteration and this recognition has significant implications (7). Hereditary kidney cancer is thought to account for 5–8% of all kidney cancer cases, although this number is likely an underestimation since a more recent study found germline mutations in up to 38% of all metastatic kidney cancer patients (6).

Mostly of the renal masses are asymptomatic until the late disease stages and are detected incidentally due to an increased use of imaging techniques for unrelated clinical issues as abdominal pain (8). The classic triad of flank pain, visible hematuria and palpable abdominal mass is rare (0.6%) (9). Up to 17% of RCCs are metastatic at diagnosis (10). Even if there is ongoing rise in incidence of RCC in early stages, mortality trends vary (11). The low prevalence of the disease does not support screening programmes which would be associated to potential false positive and over-diagnosis of slow-growing tumors (12).

The diagnosis of RCC is a challenge due to the lack of symptoms and the absence of a screening program. The use of biomarkers would be essential to improve early diagnosis and risk stratification by deepening our understanding of intratumoral biology. The development and prognosis of RCC are largely shaped by genetic alterations and dysregulation of gene expression in key pathways, including hypoxia signaling (VHL/HIF regulation), chromatin remodeling (SWI/SNF complex), and intracellular signaling cascades such as those mediated by inositol 1,4,5-triphosphate (IP3) (13). Given the critical functional impact of these gene products in cancer-associated processes such as cell proliferation and migration, identifying these biomarkers of genetic susceptibility is essential to improve early diagnosis and risk stratification by deepening our understanding of intratumoral biology. Among the biomarkers with the greatest potential are SNPs (single nucleotide polymorphisms), which allow patients to be stratified based on the study of individual alleles or in combination with other variants (14).

In hereditary RCC, germline mutations have been well-characterized in *VHL*, *MET*, and *FLCN*, associated with von Hippel–Lindau disease, papillary RCC, and Birt–Hogg–Dubé syndrome, respectively. In sporadic RCC, the recent implementation of large-scale genomic studies, such as genome-wide association studies (GWAS), has identified autosomal loci associated with increased RCC susceptibility. Among these loci, notable variants include those in *ITPR2* (rs10771279 and rs1049380) (15–19), *EPAS1* (rs7579899) (20–24), *DPF3* (rs4903064) (2, 20, 25–27), *ZEB2* (rs12105918) (16, 19, 20, 27, 28), and *PVT1/MYC* (rs35252396) (19, 20, 29, 30). *ITPR2* mediates intracellular calcium signaling from the endoplasmic reticulum to the cytosol upon IP3 binding. Variants rs10771279 and rs1049380 are in intronic and 3’UTR regions, respectively and are highly correlated with RCC susceptibility (16).

*EPAS1* encodes HIF-2α, a master regulator in the VHL/HIF hypoxia response pathway, involved in energy metabolism, angiogenesis, and apoptosis (22, 27, 31). Variant rs7579899, located in intron 1, shows again a strong correlation with RCC susceptibility.

*DPF3* is a component of the SWI/SNF chromatin remodeling complex. Variant rs4903064 is located at chr14q24.2, a regulatory region of *DPF3* that, when altered, leads to HIF-dependent expression through the creation of a novel binding site and thus functionally linking chromatin remodeling and hypoxia-driven oncogenesis (26).

*ZEB2* is a transcriptional repressor involved in key tumorigenic processes such as epithelial-to-mesenchymal transition (EMT), invasion, and immune modulation. Its expression is also HIF-regulated and significantly upregulated in RCC. rs12105918 localizes to intron 2 of the *ZEB2* gene, inside the proposed RCC-susceptibility locus 2q22.3 (20, 28, 29).

*PVT1* is a long non-coding RNA whose overexpression in RCC correlates with poor prognosis. *MYC*, a well-established proto-oncogene, regulates cell cycle, apoptosis, and genomic stability. Transactivation of both *PVT1* and *MYC* is mediated by HIF, further linking hypoxia signaling to novel tumorigenic pathways. Recent studies have shown that the rs35252396 variant alters HIF binding affinity at enhancer regions of both genes (29).

Genome-wide association studies (GWAS) have robustly identified several common genetic variants associated with sporadic renal cell carcinoma (RCC) susceptibility. However, the functional, prognostic, and population-specific relevance of many of these loci remains incompletely characterized beyond statistical association. The aim of this study was to evaluate previously GWAS-identified RCC susceptibility variants in a Spanish case-control cohort, focusing on their association with RCC risk and overall survival. Additionally, we conducted exploratory analyses integrating germline variation with tumor gene expression and multivariable risk stratification models.

## 2 Materials and methods

### 2.1 Study population

The study population consisted of a patient group (*n* = 168), recruited by urologists at the “Virgen de las Nieves University Hospital” in Granada, Spain, between 2018 and 2024. We have collected clinical data from all patients including age, sex, stage (I–IV), and metastatic status. In our study, we included patients with at least 1 year of follow-up after nephrectomy. We classified metastatic “met” patients as those who developed metastasis within 1 year of disease onset, while “non-met” refers to patients who have not yet developed metastasis. Buccal swabs were collected either on the day of surgery or during follow-up visits.

Healthy control (HC) samples (*n* = 259) were recruited from the communities of “Gran Capitán,” “Salvador Caballero,” and “Caseria de Montijo” in Granada. The control group was composed of individuals with no family history of tumors. Two buccal swabs were collected from each patient and control participant and frozen at −20 °C until processing and DNA extraction.

Table 1 summarizes the demographic and clinical characteristics of the study cohort, formalin-fixed paraffin-embedded (FFPE) tumor tissues, along with adjacent healthy tissue, were collected from a random subset of patients (*n* = 33). The study was approved by the Provincial Research Ethics Committee of Granada (CEI) under protocol code 0165-N-19. Informed written consent was obtained from all participants in accordance with the principles of the Declaration of Helsinki.

**Table 1:** Clinical characteristic of the study population.

Variables	Control (*N* = 259) *N* (%)	RCC patient (*N* = 168) *N* (%)	FFPE RCC patient (*N* = 33) *N* (%)
Age (mean ± SD)	61.27 ± 12.31	60.84 ± 12.40	61.78 ± 8.49
Sex			
Male	192 (74.1)	124 (73.8)	26 (78.8)
Female	67 (25.9)	44 (26.2)	7 (21.2)
Clear cell RCC stages			
Stage I	—	44 (26.2)	2 (6)
Stage II	—	9 (5.4)	1 (3)
Stage III	—	63 (37.5)	15 (45.5)
Stage IV	—	32 (19)	12 (36.4)
Other renal carcinomas	—	20 (11.9)	3 (9.1)
Grade			
I–II	—	84 (54.5)	12 (36.4)
III–IV	—	70 (45.5)	21 (63.6)
Metastasis at diagnosis			
No	—	137 (81.1)	20 (60.6)
Yes	—	32 (18.9)	13 (39.4)

RCC, renal cell carcinoma.

### 2.2 DNA extraction

Genomic DNA extraction from buccal swabs was performed using an organic protocol based on proteinase K and saline purification. DNA Quantifcation was performed by fluorescence using Qubit^™^ 3.0 (Invitrogen^™^ by Thermo Fisher Scientific, Waltham, MA, United States) and nanodrop 2000 system (Thermo Fisher Scientific, Waltham, MA, United States), this equipment was also used to check the 260/280 ratio as quality control. Extracted DNA was stored at −20 °C until genotyping step.

### 2.3 Genotyping

SNP selection was based on previously published GWAS that identified these variants as risk SNPs for RCC. The most relevant data were obtained from the NCBI database and GWAS Catalog, focusing on SNPs associated with RCC in studies published up to December 2024. Additionally, only SNPs with a minor allele frequency (MAF) above 10% in the Caucasian population, as reported in the Ensembl database, were considered. Based on these criteria, we selected *ITPR2* (rs1049380, rs10771279), *DPF3* (rs4903064), *EPAS1* (rs7579899), and *PVT1* (rs35252396) for the present analysis (see details of probes in Supplementary Table 1). DNA genotyping was performed using TaqMan^®^ Genotyping Master Mix (Thermo Fisher Scientific, Waltham, MA, United States). Allelic discrimination assays were carried out in a QuantStudio 6 Flex Real-Time PCR System (Applied Biosystems^™^ by Thermo Fisher Scientific, Waltham, MA, United States).

### 2.4 RNA extraction

One to four paraffin sections with a thickness of 8 μm per sample was deparaffinized with xylene prior to RNA extraction using the RNeasy FFPE kit (Qiagen, Hilden, Germany) according to the manufacturer’s recommendations. Total RNA concentrations were measured using a Nanodrop 1000 instrument (Thermo Fisher Scientific, Waltham, MA, United States).

### 2.5 Reverse transcription PCR and quantitative real-time PCR

RNA reverse transcription was implemented using PrimeScript RT Reagent Kit (Takara Bio, Japan). Quantitative polymerase chain reaction (qPCR) was performed with TaqMan^™^ gene expression assays (Thermo Fisher Scientific, Waltham, MA, United States) for: *ITPR2* (Assay ID: Hs00181916_m1), *ZEB2* (Assay ID: Hs00207691_m1), *MYC* (Assay ID: Hs00153408_m1) and *PVT1* (Assay ID: Hs00413039_m1). qPCR reactions were performed as follows: 95 °C during 10 min for enzyme activation; followed by 45 cycles of 15 s at 95 °C and 1 min at 60 °C for denaturing and annealing/extension. All samples were run in triplicates, with a NTC (non-template control) in each plate. Threshold cycles (Ct) ≥35 were considered undetermined values. mRNAs expression levels were quantified using the comparative threshold cycle method ( $2^{–\Delta \Delta C_{T}}$ ) relative to *HPRT1* (hypoxanthine phosphoribosyltransferase 1) and *GAPDH* (glyceraldehyde-3-phosphate dehydrogenase) average expression as an endogenous control. Relative quantification parameter (RQ or $2^{–\Delta \Delta C_{T}}$ ) was estimated for each case and used in statistical analysis.

### 2.6 Association analysis of SNPs with RCC risk

#### 2.6.1 Genotype association analysis

To evaluate the association between specific SNPs and susceptibility to RCC, we used the web-based tool SNPstats, applying binary logistic regression to compare healthy controls and RCC patients. Hardy–Weinberg equilibrium was first assessed in the patient group to confirm allelic independence. The analysis was conducted under codominant, dominant, and recessive genetic models, as implemented by SNPstats, without adjustment for covariates. Based on previously reported RCC GWAS loci, we evaluated the association between the selected SNPs and RCC risk. To avoid sparse genotype categories and unstable estimates, a dominant genetic model was therefore selected *a priori*, comparing carriers of the minor allele with non-carriers. This approach is consistent with previous GWAS evidence (Supplementary Table 2), indicating an association with the minor allele and allows for more robust estimation given the sample size. Codominant and recessive models were additionally analyzed as exploratory/sensitivity analyses. In the codominant model, each genotype is treated independently, allowing for distinct, non-additive risks. The dominant model groups heterozygotes and minor allele homozygotes together, assuming that a single copy of the minor allele is sufficient to influence risk. Conversely, the recessive model considers an effect only when two copies of the minor allele are present, grouping heterozygotes with major allele homozygotes. Furthermore, for each SNP and model, odds ratios, 95% confidence intervals, and *p*-values were calculated to quantify the strength and significance of the association. To account for multiple testing, Bonferroni correction was applied based on the number of SNPs analyzed (*n* = 5), resulting in a significance threshold of *α* = 0.01.

#### 2.6.2 Allelic combination analysis

Allelic combinations were analyzed using the expectation–maximization (EM) algorithm implemented in SNPstats, under assumptions of Hardy–Weinberg equilibrium. The algorithm estimated allelic combination frequencies within the cohort, and each inferred combination was modeled as a categorical variable in logistic regression to assess its relationship with RCC. Results were expressed as odds ratios, 95% confidence intervals, and *p*-values, using the most frequent allelic combination as the reference.

#### 2.6.3 Survival analysis

Survival analyses were conducted to investigate the association between the selected SNPs and clinical outcomes in RCC. Two endpoints were evaluated: total overall survival (OS) and five-year OS survival. Time-to-event was defined as the number of months from diagnosis to death. Patients were right-censored if alive at the last follow-up, lost to follow-up, or deceased from causes unrelated to RCC.

Multiple Cox proportional hazards models were fitted using the *survival* package (v3.8.3), including genotype, age group, and sex as covariates. Models were stratified by metastasis status at diagnosis to account for differences in baseline risk. The proportional hazards assumption was tested using Schoenfeld residuals (cox.zph function), and variables violating this assumption were adjusted with penalized spline terms. Separate Cox models were estimated under codominant, dominant, and recessive inheritance assumptions. Model outputs were summarized as hazard ratios (HRs) and visualized with forest plots. Adjusted survival curves were derived from Cox proportional hazard model predictions using ggadjustedcurves function from *survminer*, v0.5.1 R package. These curves represent model-based survival estimates in which censoring is accounted for during model fitting but is not explicitly displayed. Kaplan–Meier survival, cumulative hazard, and cumulative event curves were additionally generated from the observed data using *ggsurvplot* function, stratified by metastasis status at diagnosis.

#### 2.6.4 Association between SNPs and gene expression

To investigate whether the studied SNPs could act as cis-expression quantitative trait loci (cis-eQTLs), two *in silico* and experimental approaches were applied.

The first *in silico* approach involved retrieving gene expression data related to ccRCC from the TCGA database via the UALCAN web portal, focusing on the expression profiles of the genes of interest. A complementary analysis was performed using data from the GTEx database, extracting expression levels of the candidate genes in kidney tissue. In this analysis, samples were stratified according to the genotype of each SNP associated with the corresponding gene. The second approach was experimental and involved evaluating the expression levels of the candidate genes by RT-PCR in a subcohort of 33 paired FFPE tissue samples. Samples were first grouped by tissue type (tumor vs. adjacent normal), and subsequently stratified by genotype. Genotype grouping was performed under codominant, dominant, or recessive models, depending on the best fit for each SNP-gene association.

#### 2.6.5 Supervised models for tissue classification

Supervised machine learning models were implemented to evaluate the classification ability of the gene expression profiles supported by the respective genotypes, distinguishing tumor from non-tumor tissues. A custom pipeline was built using the *caret* and *pROC* packages, performing an initial data split of 70% for training/validation and 30% for testing, ensuring class balance. Within the training dataset, model validation was conducted using 100 bootstrap resamples with the ‘0.632’ bootstrap method, which reduces bias and variance in performance estimation by combining in-sample and out-of-sample accuracy. Model performance was primarily assessed by the area under the ROC curve (AUC). Five supervised learning algorithms were tested: two tree-based models (C5.0 Decision Tree and Random Forest), two generalized linear models (logistic regression and elastic net), and a support vector machines with a radial kernel. All models were tuned with a standard grid search protocol (tuneLength = 10). Performance metrics, including accuracy, recall, sensitivity, specificity, precision, and *F*_1_-score, were calculated from the corresponding confusion matrices for both training and test sets. The best-performing model in both phases was selected for variable importance analysis.

Although genotypes were identical across paired tumor and normal samples, they were included as covariates to evaluate their potential modulatory effect on gene expression differences between tissues. Accordingly, three sets of models were trained corresponding to the three genetic inheritance models.

### 2.7 Statistical analyses

Survival analyses and supervised machine learning were performed in R v4.5.1. Group comparisons of candidate gene expression were conducted in GraphPad Prism v8 using the Wilcoxon signed-rank test for paired samples. A significance level of 0.05 was set for all hypothesis testing. To account for multiple testing, Bonferroni correction was applied based on the number of SNPs analyzed (*n* = 5), resulting in a significance threshold of *α* = 0.01. For logistic regression and survival models, 95% confidence intervals were calculated along with statistical significance testing. The details of each analysis have been described in greater depth in previous sections.

## 3 Results

### 3.1 Genotyping

When analyzing SNP genotype associations under the three inheritance models, the variants rs10771279 (*ITPR2*) in the codominant and recessive models, rs4903064 (*DPF3*) in the codominant, dominant, and recessive models, and rs7579899 (*EPAS1*) in the recessive model showed nominally significant differences in genotype frequencies between RCC cases and healthy controls (Table 2). After Bonferroni correction, only the recessive model of rs10771279 remained statistically significant.

**Table 2:** Distribution of risk gene variants studied in RCC (*N* = 168) cases and healthy controls (*N* = 259).

SNPs	Genetic model	HC *N* (%)	RCC patient *N* (%)	*p*-value
rs1049380 (*ITPR2*)	Codominant			
	T/T	150 (58.6)	95 (56.5)	0.220
	G/T	85 (33.2)	65 (38.7)	
	G/G	21 (8.2)	8 (4.8)	
	Dominant			
	T/T	150 (58.6)	95 (56.5)	0.690
	G/T + G/G	106 (41.4)	73 (43.5)	
	Recessive			
	T/T + G/T	235 (91.8)	160 (95.2)	0.140
	G/G	21 (8.2)	8 (4.8)	
rs10771279 (*ITPR2*)	Codominant			
	T/T	81 (31.5)	66 (39.3)	*0.018*
	C/T	126 (49)	85 (50.6)	
	C/C	50 (19.5)	17 (10.1)	
	Dominant			
	T/T	81 (31.5)	66 (39.3)	0.097
	C/T + C/C	176 (68.5)	102 (60.7)	
	Recessive			
	T/T + C/T	207 (80.5)	151 (89.9)	**0.007**
	C/C	50 (19.5)	17 (10.1)	
rs4903064 (*DPF3*)	Codominant			
	T/T	137 (53.5)	73 (43.7)	*0.036*
	C/T	105 (41)	75 (44.9)	
	C/C	14 (5.5)	19 (11.4)	
	Dominant			
	T/T	137 (53.5)	73 (43.7)	*0.048*
	C/T + C/C	119 (46.5)	94 (56.3)	
	Recessive			
	T/T + C/T	242 (94.5)	148 (88.6)	*0.030*
	C/C	14 (5.5)	19 (11.4)	
rs7579899 (*EPAS1*)	Codominant			
	G/G	117 (45.5)	64 (38.1)	0.073
	A/G	108 (42)	70 (41.7)	
	A/A	32 (12.4)	34 (20.2)	
	Dominant			
	G/G	117 (45.5)	64 (38.1)	0.120
	A/G + A/A	140 (54.5)	104 (61.9)	
	Recessive			
	G/G + A/G	225 (87.5)	134 (79.8)	*0.032*
	A/A	32 (12.4)	34 (20.2)	
rs35252396 (*PVT1*)	Codominant			
	AC/AC	83 (32)	45 (26.8)	0.480
	AC/CG	121 (46.7)	83 (49.4)	
	CG/CG	55 (21.2)	40 (23.8)	
	Dominant			
	AC/AC	83 (32)	45 (26.8)	0.240
	AC/CG + CG/CG	176 (68)	23 (73.2)	
	Recessive			
	AC/AC + AC/CG	204 (78.8)	128 (76.2)	0.481
	CG/CG	55 (21.2)	40 (23.8)	

*p*-values <0.05 are shown in italics. *p*-values <0.01 (Bonferroni-corrected significance) are shown in bold.

In logistic regression analyses, several variants showed nominal associations with RCC risk across inheritance models (Table 3). Variant rs10771279 was associated with reduced risk, in the codominant model [OR C/C = 0.42 (0.22–0.80)] similarly, rs7579899 is associated under the recessive model [*p* = 0.033; OR = 1.78 (1.05–3.03)]; and rs4903064 (DPF3) shows nominal risk-increasing effects in recessive/codominant models [codominant OR = 2.53 (1.20–5.35); recessive OR = 2.21 (1.07–4.55)].

**Table 3:** Logistic regression analysis of RCC risk according to genotype.

SNPs	Genetic model	OR (95% CI)	*p*-value
rs1049380 (*ITPR2*)	Codominant		
	T/T	Ref.	
	G/T	1.21 (0.80–1.82)	0.392
	G/G	0.60 (0.26–1.41)	0.238
	Dominant		
	T/T	Ref.	
	G/T + G/G	1.08 (0.73–1.61)	0.705
	Recessive		
	T/T + G/T	Ref.	
	G/G	0.56 (0.24–1.29)	0.172
rs10771279 (*ITPR2*)	Codominant		
	T/T	Ref.	
	C/T	0.81 (0.53–1.24)	0.326
	C/C	0.42 (0.22–0.80)	**0.009**
	Dominant		
	T/T	Ref.	
	C/T + C/C	0.70 (0.46–1.05)	0.085
	Recessive		
	T/T + C/T	Ref.	
	C/C	0.48 (0.27–0.87)	0.479
rs4903064 (*DPF3*)	Codominant		
	T/T	Ref.	
	C/T	1.38 (0.90–2.09)	0.131
	C/C	2.55 (1.22–5.48)	*0.013*
	Dominant		
	T/T	Ref.	
	C/T + C/C	1.52 (1.02–2.27)	*0.037*
	Recessive		
	T/T + C/T	Ref.	
	C/C	2.22 (1.08–4.56)	*0.030*
rs7579899 (*EPAS1*)	Codominant		
	G/G	Ref.	
	A/G	1.17 (0.76–1.79)	0.486
	A/A	1.92 (1.08–3.39)	*0.025*
	Dominant		
	G/G	Ref.	
	A/G + A/A	1.33 (0.90–1.99)	0.151
	Recessive		
	G/G + A/G	Ref.	
	A/A	1.78 (1.05–3.02)	*0.033*
rs35252396 (*PVT1*)	Codominant		
	AC/AC	Ref.	
	AC/CG	1.27 (0.80–2.00)	0.319
	CG/CG	1.37 (0.79–2.36)	0.264
	Dominant		
	AC/AC	Ref.	
	AC/CG + CG/CG	1.29 (0.84–1.98)	0.240
	Recessive		
	AC/AC + AC/CG	Ref.	
	CG/CG	1.16 (0.73–1.84)	0.481

OR are adjusted for age and sex.

*p*-values <0.05 are shown in italics. *p*-values <0.01 (Bonferroni-corrected significance) are shown in bold.

After Bonferroni correction, only rs10771279 remained statistically significant under the recessive model.

Rather than evaluating individual polymorphisms, SNP combination analysis may provide a complementary approach for estimating RCC risk. When the analysis was restricted to the three SNPs that showed significant associations in the previous individual analyses, *ITPR2* (rs10771279), *DPF3* (rs4903064) and *EPAS1* (rs7579899), TTG was the most common allelic combination with frequencies of 26.26% in HC and 24.51% in RCC. Notably, the combination TCA was nominally significant associated with an increased odds of RCC [*p*-value = 0.035, OR = 2.28 (1.06–4.90)] However, this association did not remain statistically significant after Bonferroni correction and should therefore be interpreted as exploratory (Table 4).

**Table 4:** Association between allelic combinations and response, adjusted for age and sex.

rs10771279 (*ITPR2*)	rs4903064 (*DPF3*)	rs7579899 (*EPAS1*)	HC (frequency, %)	RCC (frequency, %)	OR (95% CI)	*p*-value
T	T	G	26.26	24.51	1.00	—
C	T	G	22.18	13.32	0.62 (0.34–1.16)	0.13
T	T	A	14.44	17.22	1.23 (0.67–2.24)	0.51
T	C	G	11.28	13.67	1.47 (0.74–2.93)	0.27
C	T	A	11.14	11.11	1.14 (0.62–2.08)	0.67
C	C	G	6.81	7.43	1.06 (0.48–2.32)	0.89
T	C	A	4.04	9.19	2.28 (1.06–4.90)	*0.035*
C	C	A	3.84	3.56	0.98 (0.33–2.88)	0.96

*p*-values <0.05 are shown in italics. None of the associations remained statistically significant after Bonferroni correction (corrected threshold: *p* < 0.0071).

Additionally, when expanding the analysis to include all five studied SNPs *ITPR2* (rs1049380, rs10771279), *DPF3* (rs4903064), *EPAS1* (rs7579899), *PVT1* (rs35252396) the most frequent combination was TTTGAG, observed in 14.95% of healthy controls and 11.47% of RCC patients. The combination TTCGAC was nominally significant associated with an increased odds of RCC, while TCTGAC appeared to have a protective effect, potentially reducing RCC susceptibility (Supplementary Table 3). However, none of these exploratory associations remained statistically significant after Bonferroni correction.

### 3.2 Survival outcomes in relation to genetic variants in RCC

**Figure 1:**
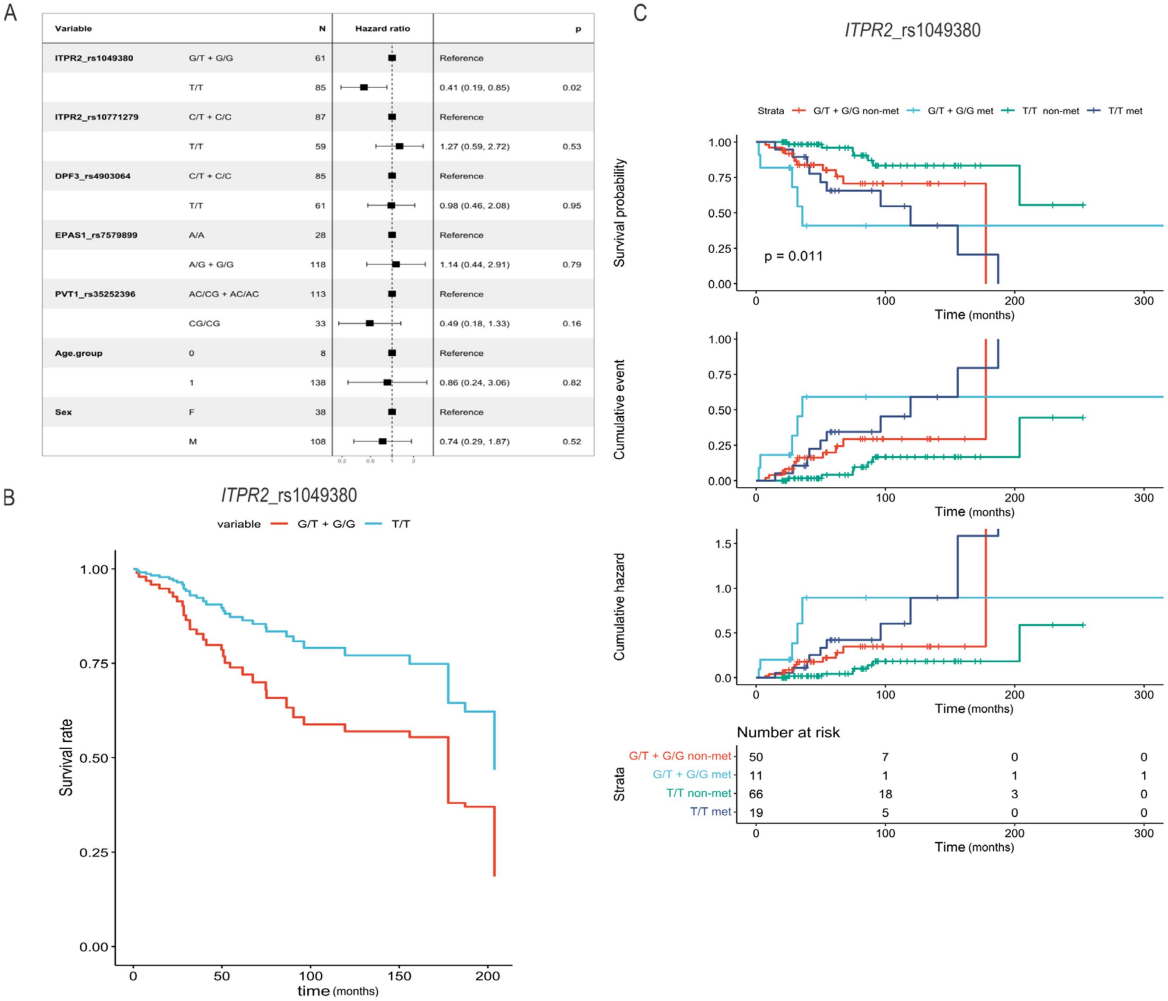
Overall survival analysis according to SNP genotypes and metastatic status in RCC patients. **(A)** Forest plot from the Cox proportional hazards regression model (dominant model). **(B)** Adjusted survival curves derived from the Cox proportional hazards model comparing overall survival between *ITPR2* rs1049380 genotypes (T/T vs. G/T + G/G). **(C)** Kaplan–Meier survival curves based on observed data for *ITPR2* rs1049380, stratified by metastasis status, showing survival probability, cumulative events, and cumulative hazard curves.

In the analysis of OS, only the dominant model for the SNP *ITPR2* rs1049380 revealed a nominal association with patient outcomes (Figure 1). Cox proportional hazards regression under a dominant model indicated that individuals with the T/T genotype exhibited a significantly reduced hazard of death compared to carriers of the G allele (G/T + G/G), with an adjusted hazard ratio (HR) of 0.41 (95% CI, 0.19–0.85, *p* = 0.02). However, this association did not remain statistically significant after Bonferroni correction for multiple testing (*α* = 0.01). To further explore this observation, adjusted survival curves derived from the Cox model were generated (Figure 1B). Kaplan–Meier survival curves, adjusted for metastasis status and other covariates, showed this trend toward improved survival for individuals with the T/T genotype, including among patients with metastasis at diagnosis. However, an inspection of the number-at-risk table over time revealed that most events occurred within the first 100 months, indicating a potential bias due to long-term survivors. To mitigate this, and considering the widespread use of 5-year overall survival as a clinical benchmark, subsequent analyses focused on evaluating the impact of all studied SNPs and clinical covariates within this 5-year window. These additional analyses aim to better assess the short- to medium-term prognostic utility of the SNPs, particularly in contemporary RCC cohorts.

**Figure 2:**
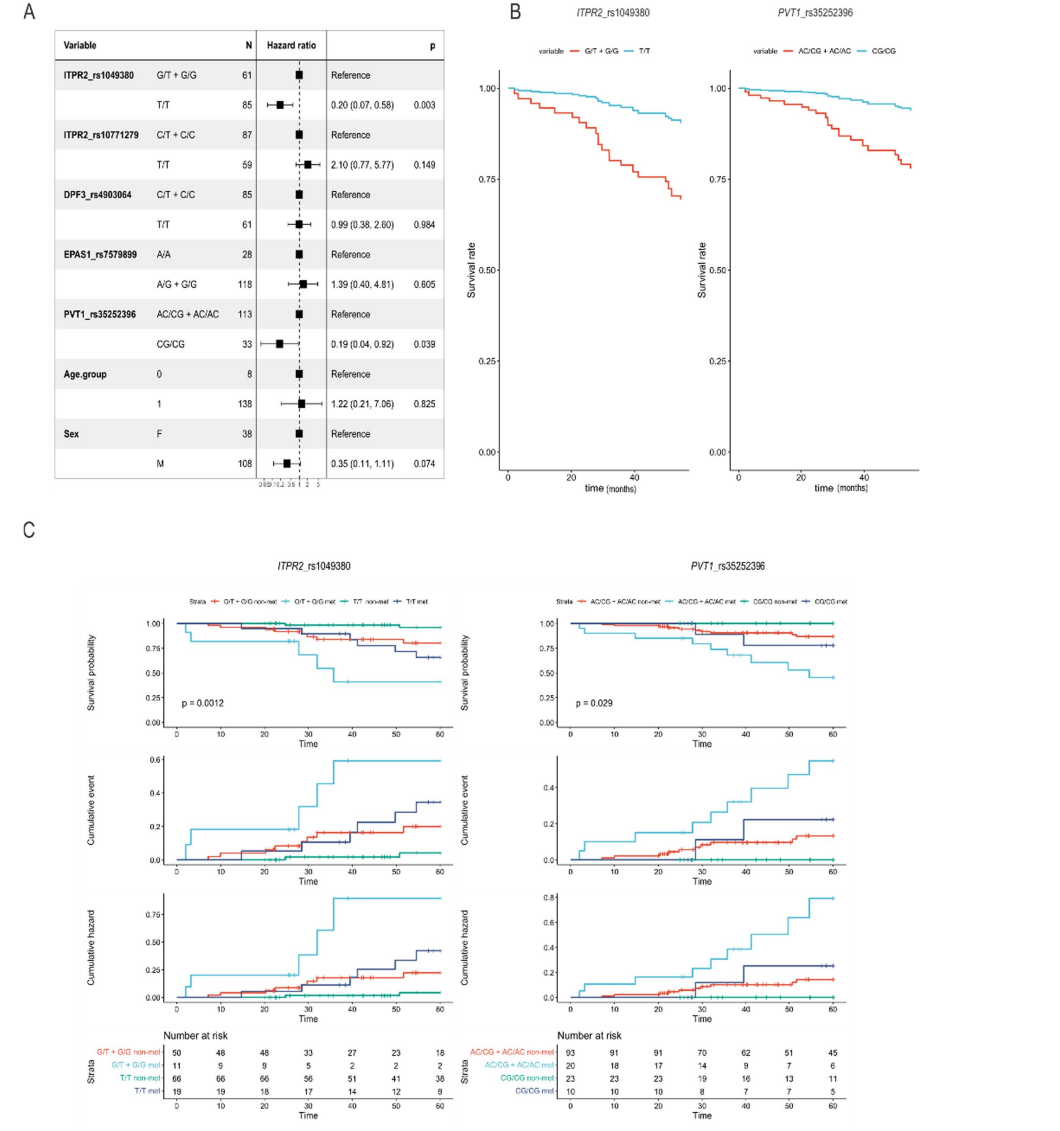
Five-year overall survival analysis according to SNPs genotypes and metastatic status in RCC patients. **(A)** Forest plot from the Cox proportional hazards regression model (dominant model). **(B)** Adjusted survival curves derived from the Cox proportional hazards model comparing five-years overall survival between *ITPR2* rs1049380 and *PVT1 rs35252396* genotypes. **(C)** Kaplan–Meier survival curves based on observed data for *ITPR2* rs1049380 and *PVT1* rs35252396 stratified by metastasis status, showing survival probability, cumulative events, and cumulative hazard curves.

Cox regression analysis identified a significant association with 5-year overall survival between *ITPR2* rs1049380 and 5-year overall survival under the dominant model (Figure 2). Individuals carrying the T/T genotype showed a reduced odds of death compared to carriers of the G allele (HR = 0.20, 95% CI: 0.07–0.58, *p* = 0.003) an association that remained statistically significant after Bonferroni correction (*α* = 0.01). For *PVT1* rs35252396, the CG/CG genotype showed a nominal association with improved 5-year survival compared with other genotypes (HR = 0.19, 95% CI: 0.04–0.92; *p* = 0.039); however, this association did not remain statistically significant after correction for multiple testing.

Kaplan–Meier survival curves illustrated these associations, and stratification by metastatic status showed a consistent direction of effect across metastatic and non-metastatic patients. The association observed for *ITPR2* rs1049380 remained statistically significant after Bonferroni correction, supporting a potential prognostic relevance. In contrast, the association observed for *PVT1* rs35252396 did not remain significant after correction and should therefore be interpreted as exploratory.

### 3.3 Differential expression genes analysis *in silico* and FFPE tissue validation

Given the reported influence of SNPs on HIF-regulated gene expression, we analyzed the expression profiles of *ITPR2*, *MYC*, *PVT1*, *ZEB2*, *DPF3*, and *EPAS1* (HIF-2α) in normal versus primary tumor tissues using the UALCAN database. We found that *ITPR2* was highly expressed in healthy tissue compared to primary tumor; and *MYC*, *PVT1*, *ZEB2*, *EPAS1* and *DPF3* were downregulated, *p*-value <0.001, (Supplementary Figure 1).

**Figure 3:**
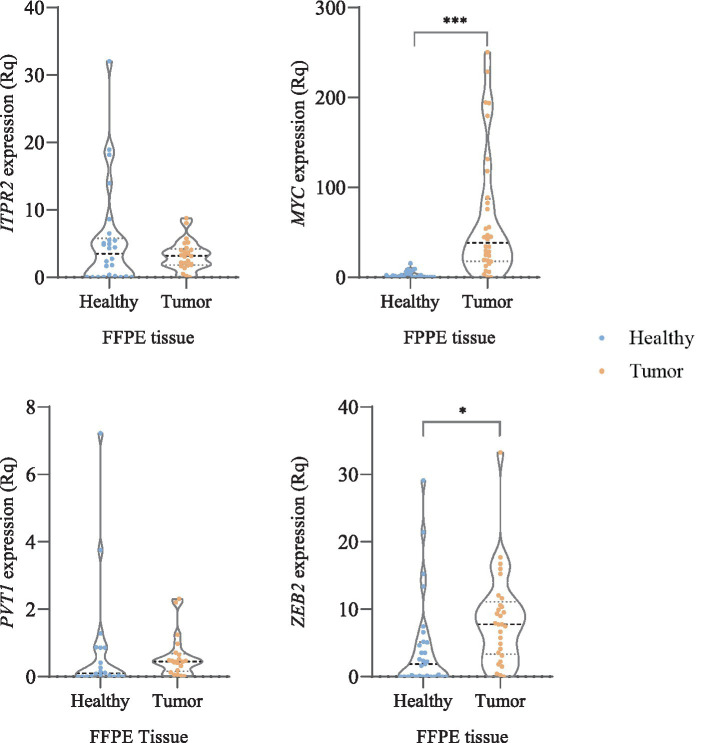
Genes expression analysis in FFPE tumor tissue and adjacent healthy tissue. * (*p*-value <0.05) and *** (*p*-value <0.001). Relative mRNA expression was quantified using the $2^{–\Delta \Delta C_{T}}$ method. Ct values ≥35 were considered undetermined. Expression was normalized to the mean of two endogenous controls (*HPRT1* and *GAPDH*).

Gene expression values for each patient were calculated as the median ± interquartile range (IQR) from three independent replicates (Supplementary Table 4). Tukey’s range test was applied to identify potential outliers. *MYC* and *ZEB2* exhibited significantly different expression patterns when comparing HC samples to RCC (*p*-value <0.001 and *p*-value = 0.012, respectively). Additionally, expression levels of *ITPR2* were higher in HC tissue compared to RCC, whereas the opposite pattern was observed for *PVT1*, with higher levels in RCC tissue than in HC (Figure 3). However, these differences were not statistically significant. With the aim of supporting the results obtained from *in silico* analyses using UALCAN and GTEx, we implemented a gene selection strategy based on supervised machine learning models to identify those candidates with the greatest predictive capacity to discriminate between FFPE tumor and non-tumor samples. In the training dataset, Random Forests consistently demonstrated the highest predictive performance across all inheritance models (codominant, dominant, and recessive), with accuracies above 0.91 and *F*_1_-scores close to 0.92 (Table 5).

**Table 5:** Performance metrics of machine learning models for RCC risk prediction (training set).

Genetic model	Model	Accuracy	Sensitivity	Specificity	Precision	Recall	*F*_1_
Codominant	Logistic regression	0.5170	0.5519	0.4826	0.5120	0.5519	0.5312
	Elastic net	0.5480	0.5344	0.5628	0.5725	0.5344	0.5528
	C5.0 Decision Tree	0.8547	0.8799	0.8264	0.8502	0.8799	0.8648
	Random Forests	0.9114	0.9021	0.9207	0.9192	0.9021	0.9106
	Radial kernel SVM	0.3483	0.3753	0.3229	0.3435	0.3753	0.3587
Dominant	Logistic regression	0.6563	0.6988	0.6166	0.6303	0.6988	0.6628
	Elastic net	0.7066	0.7425	0.6719	0.6867	0.7425	0.7135
	C5.0 Decision Tree	0.8823	0.9149	0.8493	0.8600	0.9149	0.8866
	Random Forests	0.9172	0.9021	0.9324	0.9303	0.9021	0.9160
	Radial kernel SVM	0.5767	0.5714	0.5823	0.5933	0.5714	0.5822
Recessive	Logistic regression	0.6754	0.6810	0.6697	0.6749	0.6810	0.6779
	Elastic net	0.5871	0.5516	0.6224	0.5919	0.5516	0.5711
	C5.0 Decision Tree	0.8384	0.8758	0.8009	0.8151	0.8758	0.8444
	Random Forests	0.9196	0.9068	0.9324	0.9306	0.9068	0.9185
	Radial kernel SVM	0.5222	0.5034	0.5405	0.5150	0.5034	0.5091

In the test dataset, both Random Forests and C5.0 Decision Tree models achieved perfect classification metrics across all inheritance models (accuracy, sensitivity, specificity, precision, recall, and *F*_1_-score = 1.00), which reflects strong predictive capability but may also suggest overfitting due to the small test set size. Interestingly, logistic regression and elastic net models performed better on the test set than in training, particularly under the dominant model (accuracy 0.70–0.80; *F*_1_ ≈ 0.75–0.80). The SVM model showed some improvement on the test set but remained the least effective overall (Table 6). Overall, *MYC* consistently emerged as the gene with the highest predictive relevance across the best-performing classification models, showing a greater contribution to the discrimination between tumoral and non-tumoral FFPE tissues compared to the other genes evaluated.

**Table 6:** Performance metrics of machine learning models on the test set for RCC risk prediction.

Genetic model	Model	Accuracy	Sensitivity	Specificity	Precision	Recall	*F*_1_
Codominant	Logistic regression	0.7000	0.8000	0.6000	0.6667	0.8000	0.7273
	Elastic net	0.8000	0.6000	1.0000	1.0000	0.6000	0.7500
	C5.0 Decision Tree	1.0000	1.0000	1.0000	1.0000	1.0000	1.0000
	Random Forests	1.0000	1.0000	1.0000	1.0000	1.0000	1.0000
	Radial kernel SVM	0.4000	0.6000	0.2000	0.4286	0.6000	0.5000
Dominant	Logistic regression	0.8000	0.8000	0.8000	0.8000	0.8000	0.8000
	Elastic net	0.8000	1.0000	0.6000	0.7143	1.0000	0.8333
	C5.0 Decision Tree	1.0000	1.0000	1.0000	1.0000	1.0000	1.0000
	Random Forests	1.0000	1.0000	1.0000	1.0000	1.0000	1.0000
	Radial kernel SVM	0.7000	0.4000	1.0000	1.0000	0.4000	0.5714
Recessive	Logistic regression	0.8000	0.6000	1.0000	1.0000	0.6000	0.7500
	Elastic net	0.8000	0.6000	1.0000	1.0000	0.6000	0.7500
	C5.0 Decision Tree	1.0000	1.0000	1.0000	1.0000	1.0000	1.0000
	Random Forests	1.0000	1.0000	1.0000	1.0000	1.0000	1.0000
	Radial kernel SVM	0.7000	0.6000	0.8000	0.7500	0.6000	0.6667

Random Forests and C5.0 Decision Tree emerged as the most promising classifiers for RCC risk prediction. The dominant and recessive genetic models yielded better results than the codominant model in most algorithms. Further validation using larger datasets or cross-validation is recommended to ensure generalizability and assess potential overfitting.

### 3.4 Genotype–phenotype correlation: eQTL analysis *in silico* and FFPE tissue validation

**Figure 4:**
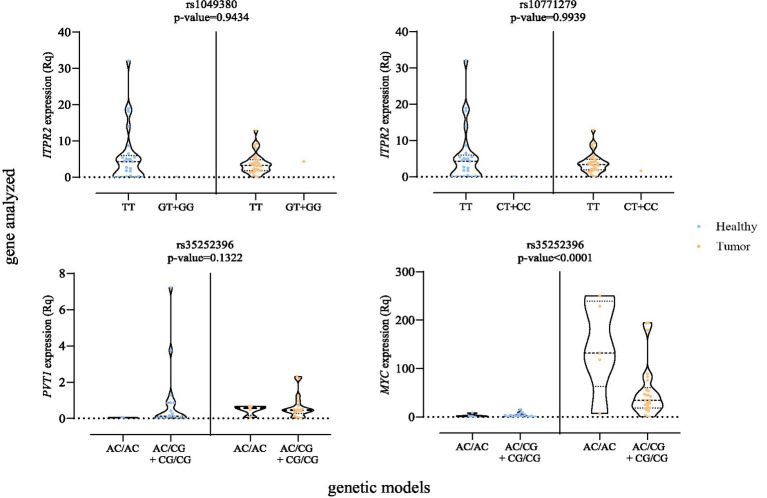
Expression levels of *ITPR2*, *PVT1*, and *MYC* genes by genetic model in tumor and adjacent healthy FFPE tissues.

The exploratory analysis conducted using UALCAN prompted further investigation into the impact of SNPs on the expression of *ITPR2*, *PVT1* and *MYC*, given the non-coding position of the studied SNPs and the potential additional impact that alterations in these genes may have on multiple oncogenic pathways. Our eQTL analysis was conducted both *in silico* using the GTEx platform and experimentally on FFPE tissue samples. *In silico* analysis revealed that among studied SNPs, only rs1049380 (*ITPR2*) showed a significant eQTL association specifically in kidney cortex, while no significant associations were observed in the whole blood (Supplementary Figure 2). However, due to the absence of expression data for *MYC* and *PVT1* in GTEx, the analysis remains limited in scope. To address this limitation, we further evaluated gene expression by stratifying FFPE samples according to genotype groups for rs1049380 (*ITPR2*, the SNP with *in silico* significant, as well as rs35252396, associated with both *PVT1* and *MYC*). Specifically, For FFPE tissue samples, *ITPR2*, *MYC* and *PVT1* eQTL analyses were performed using the most significant genetic models, with tissue separated into tumor and adjacent healthy tissue area. Only *MYC* expression yielded statistically significant results (*p*-value <0.001) (Figure 4).

Notably, both rs1049380 (*ITPR2*) and rs10771279 (*ITPR2*) in kidney tissue followed the expression trends described in GTEx; however, these differences did not reach statistical significance in our FFPE analyses. Furthermore, the expression of *PVT1* and *MYC* was analyzed in relation to rs35252396. To the best of our knowledge, this represents the first study to evaluate this eQTL in human kidney tissue. Interestingly, higher *MYC* expression levels were observed in individuals carrying CG allele, although these differences did not reach statistical significance (Supplementary Figure 2).

## 4 Discussion

Significant efforts have been focused on evaluating *ITPR2* (rs1049380, rs10771279), *DPF3* (rs4903064), *EPAS1* (rs7579899), and *PVT1* (rs35252396) variants in relation to RCC susceptibility. In the kidney, receptors such as vasopressin, endothelin, and angiotensin II are linked to phosphatidylinositol signaling. Previous studies have shown that *ITPR2* is the most abundantly expressed subtype in murine kidney tissue, surpassing *ITPR1*, while *ITPR3* is undetectable. Exposing mice to nephrotoxic agents lead to significantly elevated mRNA and protein levels of *ITPR2* in the kidney, without affecting *ITPR1* levels. Suggesting that *ITPR2* may play an important role in kidney function; and may be involved in induced nephrotoxicity (32). In line with these observations, *ITPR2* expression levels in our cohort were higher in healthy kidney tissue than in tumor tissue, supporting the biological plausibility of altered calcium signaling in RCC development (16). By contrast, in a separate microarray gene expression study, *ITPR2* was among the most significantly downregulated genes in RCC tumoral tissue compared to adjacent healthy kidney (33). In this study we focus on rs10771279, an intronic variant located at 12p11.23 in *ITPR2*, positioned 77 kb from the European-ancestry RCC risk marker rs718314 which was strongly associated with RCC (*p*-value = 1.2 × 10^–7^) (16). Although the association observed for rs10771279 did not remain statistically significant after Bonferroni correction, our results suggest that C/C genotype of may have a protective role in RCC patients, this finding is directionally consistent with the study by Purdue et al. (16); being T allele a risk factor for RCC.

We additionally evaluated rs1049380 in *ITPR2* in relation to disease risk and survival outcomes. In contrast to rs10771279, no association was observed between rs1049380 and RCC susceptibility in case-control analyses, either before or after multiple-testing correction. However, a consistent association with survival outcomes was identified. Previous studies have linked rs1049380 mainly to increased risk of RCC and younger age at diagnosis rather than survival outcomes, with the A (risk) allele being more frequent in RCC cases compared with controls (17). While prior GWAS consistently identify A as the susceptibility allele (17), our cohort shows T as the risk-increasing allele—a discrepancy potentially due to strand orientation differences, population-specific LD patterns, or cohort selection effects. To date, no published work had established a prognostic impact of this variant; thus, our findings may suggest a potential prognostic role of rs1049380, with the G allele being associated with poorer survival. This association was observed for both overall survival and 5-year overall survival; however, only the 5-year survival analysis remained statistically significant after Bonferroni correction, suggesting a time-dependent effect, with the strongest impact occurring during the early to medium follow-up period. Such context-dependent effects are consistent with the involvement of *ITPR2* in calcium signaling pathways, possibly through alterations in *ITPR2*-mediated calcium signaling that may influence tumor cell proliferation and apoptosis (39).

In the present study, we observe nominal association between individuals carrying the AA genotype in rs 7579899 (*EPAS1*) variant and RCC risk. Although this association did not remain statistically significant after Bonferroni correction, the direction of effect is consistent with previous GWAS, which have identified the A allele as the RCC risk allele (23). *EPAS1* is well-known to be dysregulated in RCC due to the loss of *VHL* protein function, serving as the primary driver of RCC and regulating multiple critical oncogenic pathways (34). The SNP rs7579899 is located within *EPAS1*, the gene encoding *HIF*-2α, a critical mediator of hypoxia signaling in the HIF/VHL pathway implicated in RCC pathogenesis (20). Loss of pVHL leads to HIFα accumulation and activation of pro-tumorigenic genes, with HIF2α playing a key role in tumor growth and early cellular atypia in renal lesions (21).

*DPF3* is a component of the SWI/SNF chromatin remodeling complex and has been associated with RCC susceptibility in previous GWAS. rs4903064, was identified as a risk variant for RCC (27). In our cohort, we observed a nominal association between rs4903064 and RCC risk, with individuals carrying the CC genotype showing an increased risk of disease. Although this association did not remain statistically significant after Bonferroni correction, the direction of effect is consistent with GWAS evidence, which identifies the C allele as the risk allele (27).

It has been shown that the C risk allele creates a hypoxia-inducible binding site that may act as an activator in RCC development. Stratification of individuals in an RCC cohort based on rs4903064 genotype revealed significantly higher *DPF3* mRNA levels in tumors from carriers of at least one C allele, indicating that this SNP may reside within a regulatory DNA element that functions as an enhancer of *DPF3* expression (25, 26). In motif analyses, rs4903064-T creates a binding site for IRX2/IRX5, while the C risk allele creates a binding site for *HIF*. IRX2 and IRX5 are known transcriptional repressors, while *HIF* can be an activator, which is consistent with the observed stronger allelic effect under hypoxia (26). Recent earlier studies have reported that *DPF3* overexpression could increase growth rate in kidney cell lines (25), whereas genetic knockout of *DPF3* in human urinary primary tubular cells significantly decreased cell proliferation *in vitro* (26). Cui et al. (35) demonstrated that *DPF3* promoted kidney cancer cell migration *in vitro* and *in vivo* revealing the mechanism underlying *DPF3*-mediated gene regulation. *DPF3* enhances kidney cancer cell migration and metastasis by interacting with key regulatory proteins like SNIP1, SMAD4, and p300 histone acetyltransferase, which are involved in the TGF-β signaling (36). This interaction leads to the activation of genes related to cell movement, such as *SNAI1* and *MMP2*, by increasing histone acetylation and activating the transcription of target genes (35). Collectively, these findings provide strong biological plausibility for the involvement of rs4903064 in RCC pathogenesis, despite the exploratory nature of the association observed in our cohort.

The polymorphism rs35252396 lies in an intergenic, putative regulatory region between *MYC*, a major oncogenic driver, and *PVT1*. *MYC* is a key regulator of metabolic and growth-promoting pathways, and its dysregulation is a hallmark of tumor initiation. Although this variant (rs35252396) has been identified as a risk allele (CG) associated with RCC in previous GWAS (30). In our cohort, rs35252396 was not associated with RCC risk in case-control analyses. However, we observed a nominal association with survival outcomes, suggesting a potential role in disease progression rather than cancer susceptibility. Although this association did not remain statistically significant after Bonferroni correction, the direction of effect was consistent with GWAS, with carriers of the CG risk allele showing poorer survival.

Present data of expression analysis of *MYC* and *PVT1* in FFPE RCC tumor and adjacent HC tissue revealed significantly higher expression levels in tumor regions compared to adjacent normal tissue. Findings which are consistent with previous reports indicating co-amplification and overexpression of both genes in RCC tissue. In tumor tissue, *PVT1* RNA levels strongly correlate with *MYC* expression; and appear to stabilize *MYC* protein, thereby promoting tumor growth. These observations suggest a functional interplay between *MYC* and *PVT1* that is critical for oncogenesis at the 8q24 locus (29).

Bioinformatic analyses have reported elevated *ZEB2* expression in RCC compared to normal kidney tissue, highlighting its potential role in promoting EMT and its involvement in *anoikis* evasion, a crucial step for metastasis (37). Our findings corroborate this data, showing that *ZEB2* expression is significantly higher in RCC compared to HC, further supporting its oncogenic potential in this context (38).

One of the main limitations of this study is the relatively small sample size, particularly in the subgroup analyses involving gene expression. The limited number of cases may reduce statistical power and limit the generalizability of the findings. Regarding our machine learning approach, we now state that the classification models were not intended to provide a generalized predictor of RCC, but rather to support a gene selection strategy based on expression-driven discriminative capacity. This approach allowed us to highlight the relevance of *MYC* in distinguishing tumoral from non-tumoral FFPE samples and to prioritize candidate genes for genotype-based eQTL exploration, complementing external evidence from UALCAN and GTEx with data derived from our own cohort. Future studies involving larger and more diverse cohorts are warranted to validate and expand upon these results.

## 5 Conclusion

This study highlights the contribution of specific SNPs to the risk and prognosis of RCC. Regarding RCC risk, nominal associations were observed for *ITPR2* rs10771279 and *EPAS1* rs7579899 in our Spanish cohort, with effect directions consistent with previous GWAS findings. In contrast, survival analyses identified *ITPR2* rs1049380 as a variant of potential prognostic relevance, as the association with five-year overall survival remained statistically significant after Bonferroni correction. Additional exploratory associations with survival were observed for *PVT1* rs35252396, although these did not survive multiple-test correction. Furthermore, expression analyses revealed increased *MYC* and *PVT1* expression in RCC tumor tissue compared with adjacent normal kidney tissue, supporting a functional role of the 8q24 locus in tumor biology. Overall, our findings suggest that distinct genetic variants may differentially influence RCC susceptibility and disease progression, and they highlight the biological plausibility of GWAS-identified loci in RCC.

## Data Availability

The datasets (excel sheets with per sample genotype, clinical, survival, and RT-qPCR expression values) are available in Zenodo (DOI:10.5281/zenodo.18600365) under restricted access upon request, and the R scripts used for survival and machine learning analyses are publicly available at https://github.com/fmarinb/RCC_SNP_validation_survival_ml.
