# Supplementary material for: Validation of ITPR2, DPF3, EPAS1, and PVT1-associated SNPs as biomarkers for RCC in an independent case-control cohort
Source: Front Med (Lausanne). 2026 Mar 11;13:1734511. doi: 10.3389/fmed.2026.1734511 (PMC13012921; doi:10.3389/fmed.2026.1734511)
Supplement: Supplementary file 1 [file Data_Sheet_1.docx]

***Supplementary***

**Supplementary Table 1.** Genomic and functional annotation of the analyzed SNPs.

| **SNPs** | **Location** | **Consequence** | **Allele frecuency** | **Target Sequence** |
| --- | --- | --- | --- | --- |
| rs1049380 (*ITPR2*) | Chromosome 12:26336611 (forward strand) | 3 prime UTR variant | T: 0.771  G: 0.229 | GTTATTTTAACTCAGAAAACATACT[G/T]GCATTAAGCTCTTGAGCCTCAGAAT |
| rs10771279 (*ITPR2*) | Chromosome 12:26377610 (forward strand) | intron variant | T: 0.589  C: 0.411 | CCTGTTCTCCAATACTTGATTTACA[C/T]ATATTTAGCCTTTGTCAAATTTTAC |
| rs4903064 *(DPF3*) | Chromosome 14:72812712 (forward strand) | intron variant | T: 0.790  C: 0.210 | GGGCGCTCTAGTATGCCACTTGTCA[C/T]GTACACTTTTTCTGCTGACACAGAG |
| rs7579899 (*EPAS1*) | Chromosome 2:46310465 (forward strand) | intron variant | G: 0.626  A: 0.374 | AACTGTTCATTGCACACCCTGTACA[A/G]AGCACTGCGACCAAGCTGTTCCTTG |
| rs35252396 (*PVT1*) | Chromosome 8:127877125-127877126 (forward strand) | regulatory region variant | AC: -  CG: - | TGAGTAGAGACCTGCTTCTGAGAAAGTCAGAAGTGCATTCCAAAGTCAGGAGCGTGACCTCCCCGGAAGCCCAGCTAATCCAGGTTTTAAAGAGGAGTTGGAACAAAGGTGGTGCTTATGGGATGTATGGAGCTGTGGGATGTGGGAGAGGACAGGTTTAGCAGAGTGGCGTGGTGGAAAGTTTCCTCTCGCCGAGGAGG[AC/CG]AACTCCCCAGCGCCTGAGACAGCGGGAGCTCAGAGCGCCTAGAATGTGCCCAGATTCTAAAGTGAAGCAGGCTGTGGTGTGATGATGGCCCCCGAGGGGACTTGCAGTCTTATGGACCTGGGTTCAAGGCCACCCTCTTCAGCCACACAAACTGGGCAAGGGGCCCGCCCTTTTCACGCTGCTGTTTCTGTAACTTTCAG |
| rs12105918  *(ZEB2)* | Chromosome 2:144450626 (forward strand) | intron variant | T: 0.939  C: 0.061 | AATAAGAATCATGCAAAGAAGTGTG[C/T]GGCTTACAACTGCATGTTACTTATA |

(r^2^= 0.104624; D’=0.496038; rs1049380 and rs10771279)

**Supplementary Table 2.** GWAS-reported risk alleles and direction of genetic effects on RCC susceptibility.

**.**

| **SNP ID** | **Ensembl** | | | **GWAS Catalog** | | | | | | | | | |
| --- | --- | --- | --- | --- | --- | --- | --- | --- | --- | --- | --- | --- | --- |
|  | **Population** | **Alelle Frequency** | **Genotype Frequency** | **Risk allele** | **P value** | **OR (96% CI)** | **Discovery cohort** | | | | | | **Reference** |
| **rs1049380** | EUR | G:0.271 (273)  T:0.729 (733) | G\|G: 0.070 (35)  G\|T: 0.404 (203)  T\|T: 0.527 (265) | NA | 2 x 10-12 | 1.15 [1.11-1.19] | 10,784 European ancestry cases,  20,406 European ancestry controls | | | | | | (Scelo et al. 2017) |
|  | IBS | G: 0.229 (49)  T: 0.771 (165) | G\|G: 0.065 (7)  G\|T: 0.327 (35)  T\|T: 0.607 (65) | NA | 7 x 10-10 | 1.27 [1.17-1.37] | 3,227 European ancestry male cases,  4,916 European ancestry male controls | | | | | | (Laskar et al. 2019) |
| **rs10771279** | EUR | C: 0.432 (435)  T: 0.568 (571) | C\|C: 0.173 (87)  C\|T: 0.519 (261)  T\|T: 0.308 (155) | C | 1.2 × 10−7 | 0.48 [0.36-0.63] | 843 European descent,  358 African American cases, | | | 707 European descent, 519 African American | | | (Purdue et al. 2013) |
|  | IBS | C: 0.411 (88)  T: 0.589 (126) | C\|C: 0.168 (18)  C\|T: 0.486 (52)  T\|T: 0.346 (37) |  |  |  |  |  |  |  |  |  |  |
| **rs4903064** | EUR | C: 0.242 (243)  T: 0.758 (763) | C\|C: 0.050 (25)  C\|T: 0.384 (193)  T\|T: 0.567 (285) | C | 2 x 10-24 | 1.21 [1.16-1.25] | 10,784 European ancestry cases,  20,406 European ancestry controls | | | | | | (Scelo et al. 2017) |
|  |  |  |  | C | 9 x 10-54 | 1.27[1.23-1.31] | 752,817 European ancestry individuals, | | 3,526 African American or Afro-Caribbean individuals, | | 3,457 Hispanic or Latin American individuals | | (Purdue et al. 2024) |
|  | IBS | C: 0.210 (45)  T: 0.790 (169) | C\|C: 0.028 (3)  C\|T: 0.364 (39)  T\|T: 0.607 (65) | C | 2 x 10-59 | 1.22[1.19-1.25] | 25,890 European ancestry cases, 743,585 European ancestry controls, | 897 African American or Afro-Caribbean cases, 3,109 African American or Afro-Caribbean controls, | | 1,612 Hispanic or Latin American cases,  2,180 Hispanic or Latin American controls, | | 621 East Asian ancestry cases,  86,796 East Asian ancestry controls | (Purdue et al. 2024) |
|  |  |  |  | C | 2 x 10-14 | 1.38[1.27-1.50] | 1,992 European ancestry female cases,  3,095 European ancestry female controls | | 1,612 Hispanic or Latin American cases,  2,180 Hispanic or Latin American controls, | | 621 East Asian ancestry cases, 86,796 East Asian ancestry controls | | (Laskar et al. 2019) |
| **rs7579899** | EUR | A: 0.384 (386) G: 0.616 (620) | A\|A: 0.141 (71)  A\|G: 0.485 (244)  G\|G: 0.374 (188) | A | 5 x 10-15 | 1.15[1.11-1.20] | 10,784 European ancestry cases,  20,406 European ancestry controls | | | | | | (Scelo et al. 2017) |
|  | IBS | A: 0.374 (80)  G: 0.626 (134) | A\|A: 0.150 (16) A\|G: 0.449 (48)  G\|G: 0.402 (43) | A | 1 x 10-6 | 1.15[1.10-1.21] | 3,227 European ancestry male cases,  4,916 European ancestry male controls | | | | | | (Laskar et al. 2019) |
|  |  |  |  | A | 2 x 10-9 | 1.19[1.12-1.26] | 3,772 European ancestry cases,  8,505 European ancestry controls | | | | | | (Purdue et al. 2011) |
| **rs35252396** | NA | | | CG | 5 x 10-11 | 1.27 [1.18-1.37] | 1,505 European ancestry cases,  67,725 European ancestry controls | | | | | | (Gudmundsson et al. 2013) |

**Supplementary Table 3.** Association between allelic combinations and response adjusted for age and sex, including all studied SNPs.

| **rs1049380**  **(*ITPR2*)** | **rs10771279**  **(*ITPR2*)** | **rs490306**  **(*DPF3*)** | **rs7579899**  **(*EPAS1*)** | **rs3525239**  **(*PVT1*)** | **HC**  **(Freq, %)** | **RCC**  **(Freq, %)** | **OR (95% CI)** | **p-value** |
| --- | --- | --- | --- | --- | --- | --- | --- | --- |
| T | T | T | G | AC | 14.95 | 11.47 | Ref. | - |
| T | T | T | G | CG | 7.92 | 7.58 | 1.33 (0.49 - 3.60) | 0.58 |
| T | T | T | A | CG | 5.14 | 1.06 | 2.59 (1.10 - 6.07) | *0.03* |
| T | C | T | G | CG | 7.92 | 4.3 | 0.73 (0.27 - 1.99) | 0.54 |
| T | T | C | G | AC | 3.38 | 9.94 | 4.94 (1.45 - 16.84) | *0.011* |
| T | C | T | G | AC | 8.01 | 1.84 | 0.18 (0.03 - 0.99) | *0.05* |
| T | T | T | A | AC | 4.95 | 4.7 | 0.93 (0.28 - 3.12) | 0.91 |
| G | C | T | G | AC | 4.58 | 4.7 | 1.65 (0.52 - 5.24) | 0.4 |
| T | C | T | A | CG | 4.23 | 4.73 | 1.35 (0.44 - 4.19) | 0.6 |
| T | T | C | G | CG | 4.26 | 3.68 | 0.76 (0.18 - 3.17) | 0.71 |
| G | T | T | A | AC | 3.78 | 2.54 | 0.79 (0.22 - 2.85) | 0.72 |
| G | T | T | G | CG | 4.26 | 3.68 | 1.04 (0.27 - 4.02) | 0.95 |
| T | T | C | A | AC | 3.29 | 3.15 | 1.25 (0.37 - 4.17) | 0.72 |
| T | C | T | A | AC | 3.34 | 2.26 | 1.24 (0.33 - 4.60) | 0.75 |
| T | C | C | A | AC | 2.43 | 3.12 | 1.25 (0.35 - 4.48) | 0.73 |
| T | T | C | A | CG | 1.2 | 3.73 | 4.16 (1.09 - 15.95) | *0.038* |
| G | C | C | G | CG | 2.95 | 2.01 | 0.70 (0.17 - 2.91) | 0.62 |
| G | C | T | G | CG | 1.55 | 2.94 | 1.50 (0.25 - 9.20) | 0.66 |
| G | T | C | G | AC | 2.35 | 1.17 | 0.92 (0.18 - 4.82) | 0.92 |
| T | C | C | G | CG | 1.33 | 3.07 | 1.82 (0.39 - 8.44) | 0.44 |
| G | C | T | A | CG | 2.28 | 1.64 | 1.02 (0.18 - 5.68) | 0.98 |
| G | C | T | A | AC | 1.31 | 2.36 | 2.11 (0.34 - 13.24) | 0.42 |
| T | C | C | G | AC | 2.53 | 1.73 | 0.84 (0.11 - 6.13) | 0.86 |
| Global allelic combination p-value: 0.02 | | | | | | | | |

**ORs and 95% CIs were estimated using binary logistic regression models. P-values < 0.05 indicate nominal significance. None of the associations remained statistically significant after Bonferroni correction.**

**Supplementary Table 4.** Comparison of gene expression in healthy and tumor FFPE tissues.

| **Gene** | **Healthy FFPE tissue**  **(Rq median; IQR)** | **Tumor FFPE tissue**  **(Rq median; IQR)** | **p-value** |
| --- | --- | --- | --- |
| *ITPR2* | 3.522 (0.088-5.793) | 3.168 (1.891-4.214) | 0.264 |
| *MYC* | 1.662 (0.016-4.029) | 38.31 (17.87-87.40) | **<0.001** |
| *PVT1* | 0.105 (0.008-0.865) | 0.453 (0.161-0.685) | 0.521 |
| *ZEB2* | 1.896 (0.050-5.143) | 7.792 (3.357-11.130) | **0.012** |


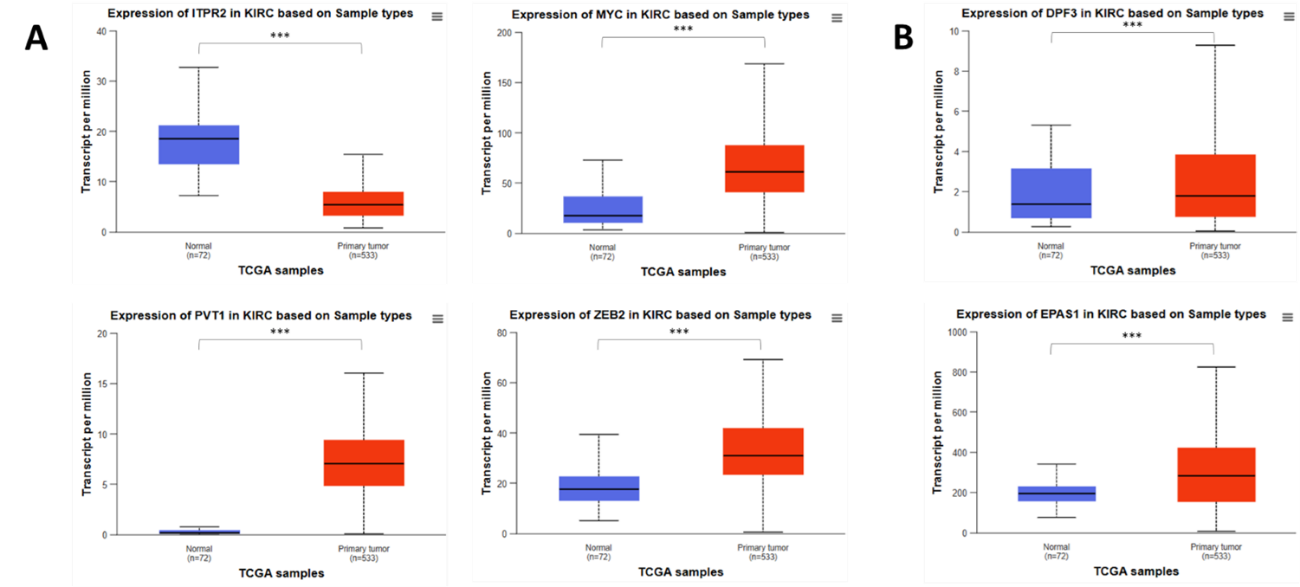
**Supplementary Figure 1.** Differential Gene Expression in RCC Tissues: Insights from UALCAN. (A) Genes analyzed in FFPE RCC samples, comparing expression levels in tumor and adjacent healthy tissues. (B) Genes selected based on SNP analysis and studied *in silico* to explore their potential role in RCC pathogenesis.


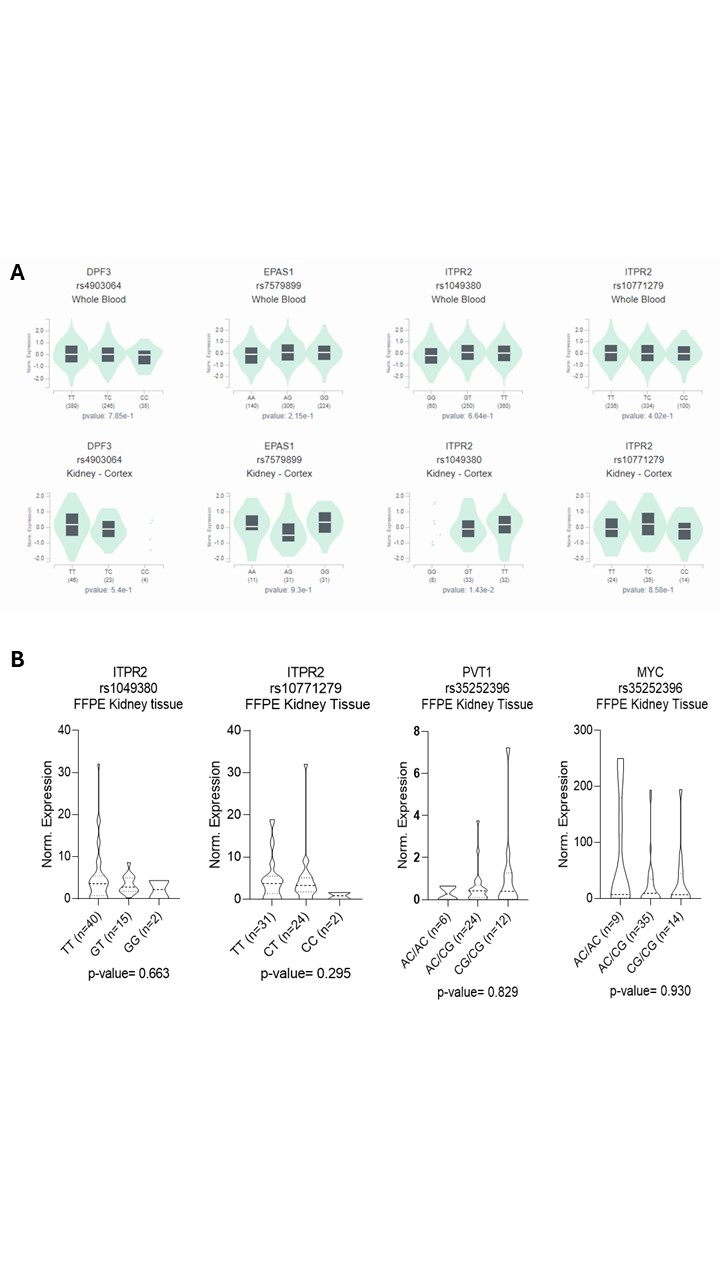
**Supplementary Figure 2.** **(A)** Tissue-Specific eQTL Analysis from GTEx Data. **(B)** FFPE Kidney tissue eQTLs analysis, including healthy and adjacent tumor tissue samples.

**Supplementary References**

Gudmundsson J, Sulem P, Gudbjartsson DF, et al (2013) A common variant at 8q24.21 is associated with renal cell cancer. Nat Commun 4:. https://doi.org/10.1038/NCOMMS3776

Laskar RS, Muller DC, Li P, et al (2019) Sex specific associations in genome wide association analysis of renal cell carcinoma. Eur J Hum Genet 27:1589–1598. https://doi.org/10.1038/S41431-019-0455-9

Purdue MP, Dutta D, Machiela MJ, et al (2024) Multi-ancestry genome-wide association study of kidney cancer identifies 63 susceptibility regions. Nat Genet 56:809–818. https://doi.org/10.1038/S41588-024-01725-7

Purdue MP, Johansson M, Zelenika D, et al (2011) Genome-wide association study of renal cell carcinoma identifies two susceptibility loci on 2p21 and 11q13.3. Nat Genet 43:60–65. https://doi.org/10.1038/NG.723

Purdue MP, Ye Y, Wang Z, et al (2013) A genome-wide association study of renal cell carcinoma among African Americans. Cancer Epidemiol Biomarkers Prev 23:209. https://doi.org/10.1158/1055-9965.EPI-13-0818

Scelo G, Purdue MP, Brown KM, et al (2017) Genome-wide association study identifies multiple risk loci for renal cell carcinoma. Nature Communications 2017 8:1 8:1–9. https://doi.org/10.1038/ncomms15724
